# Comorbidity of inflammatory bowel disease with atypical hemolytic uremic syndrome in pediatric patients 

**DOI:** 10.5414/CNCS109511

**Published:** 2019-06-25

**Authors:** H. Stella Shin, Carla M. Nester, Bradley P. Dixon

**Affiliations:** 1 Division of Nephrology, Department of Pediatrics, Emory University School of Medicine, Atlanta, GA,; 2 Division of Nephrology, Hypertension and Dialysis, University of Iowa Children’s Hospital, Iowa City, IA, and; 3 Renal Section, Department of Pediatrics, University of Colorado School of Medicine, Aurora, CO, USA

**Keywords:** inflammatory bowel disease, atypical hemolytic uremic syndrome, complement

## Abstract

Atypical hemolytic uremic syndrome (aHUS) is a form of thrombotic microangiopathy mediated by dysregulation of the alternative complement pathway. Complement-amplifying conditions such as respiratory and gastrointestinal infections, pregnancy, malignancy, and systemic autoimmune diseases such as systemic lupus erythematosus have been associated with the clinical manifestation of aHUS. Inflammation of the gastrointestinal tract is a potent stimulus for complement activation, and we describe a series of three pediatric patients with aHUS and comorbidity of inflammatory bowel disease (IBD). In two of the three cases, the diagnosis of aHUS preceded the diagnosis of IBD, perhaps suggesting a mechanistic link between complement dysregulation and thrombotic microangiopathy in the gastrointestinal tract and the ensuing inflammatory changes of IBD.

## Introduction 

Hemolytic uremic syndrome (HUS) is characterized by a clinical triad of microangiopathic hemolytic anemia, thrombocytopenia, and end-organ dysfunction, most notably acute kidney injury (AKI). The majority of HUS cases occur as a result of infection with Shiga-like toxin producing *E. coli* (STEC-HUS) but 5 – 10% of cases are due to a subtype known as atypical hemolytic uremic syndrome (aHUS). A number of these cases are caused by uninhibited activation of the alternative complement pathway [1, 2]. 

Atypical hemolytic uremic syndrome can be familial or sporadic, and various mutations in genes of the complement system have been described in some of these patients [3, 4, 5]. In 50 – 80% of pediatric patients with aHUS, complement activation is triggered by an infectious event, most commonly respiratory or gastrointestinal infections [6]. Other processes may stimulate complement activation, including pregnancy, surgery, and autoimmune disorders, leading to clinical manifestations in patients with underlying defects in complement regulation [5, 7]. Less well described is an association between inflammatory bowel disease (IBD) and aHUS. There have been only six cases of HUS associated with IBD reported in the literature, nearly all of which were in adult patients [8, 9, 10, 11, 12]. We evaluated the genetic background of three cases of pediatric patients with co-existing aHUS and IBD to determine if there was a genetic predisposition of their aHUS and whether there were any correlations between aHUS and IBD. 

## Case histories 

### Case 1 

A 13-year-old Hispanic male with a history of ulcerative colitis, diagnosed 2 years previously and marginally controlled on 6-mercaptopurine, presented to the emergency department with 1 week of abdominal pain, vomiting, and worsening hematochezia. He was found to have significant anemia (Hgb of 3.9 g/dL), thrombocytopenia (platelet count of 54 × 10^9^/L), and acute kidney injury (AKI) (serum creatinine of 1.8 mg/dL, BUN of 25 mg/dL) on presentation. His serum C3 level was normal and his serum C4 level was slightly low at 13.0 mg/dL. 

**Figure 1.**
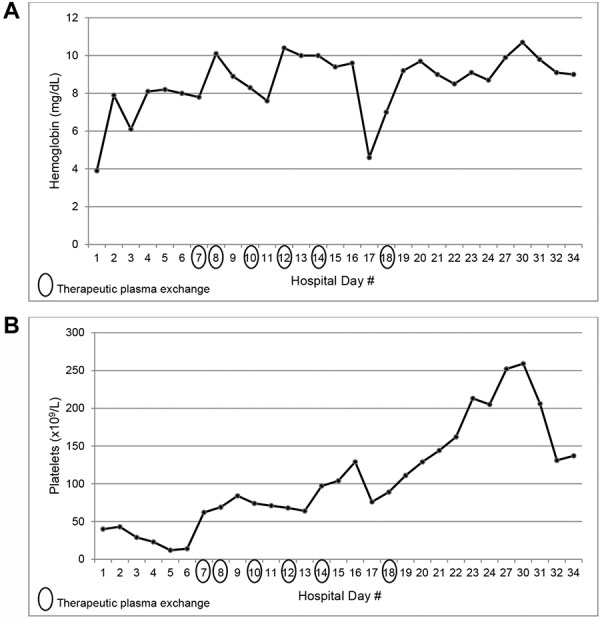
Trends in (A) hemoglobin and (B) platelet count in case 1.

The anemia, thrombocytopenia, and AKI were initially attributed to his ongoing gastrointestinal bleeding and acute tubular necrosis, but the hematologic abnormalities were refractory to 9 units of packed red blood cells and 3 units of platelets given during the first 6 days of admission. Stool culture obtained at time of presentation was negative for organisms producing Shiga-like toxin. Further workup revealed an elevated lactic acid dehydrogenase (LDH) (818 units/L), a low haptoglobin (< 6 mg/dL), normal ADAMTS13 activity level (68%), positive direct Coombs test, and presence of platelet autoantibodies. Given the presumed autoimmune etiology of the hematologic abnormalities, the patient was treated with 1 dose of 1 g/kg of intravenous immunoglobulin (IVIg) and then placed on 30 mg twice daily of methylprednisolone for 7 days, followed by an oral prednisone taper. However, his hemoglobin and platelet count continued to remain low with this therapy, and the patient subsequently received 6 treatments of therapeutic plasma exchange (TPE), over the course of which his hemoglobin and platelet count stabilized and slowly recovered to normal (Figure 1). 

**Figure 2.**
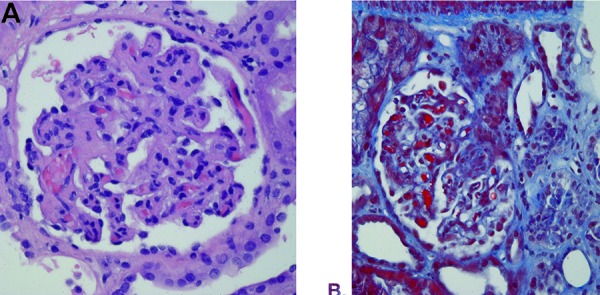
Thrombotic microangiopathy with glomerular capillary thrombi and fragmented red blood cells as noted on renal biopsy in case 1.

However, his kidney function continued to worsen, with serum BUN and creatinine further increasing to 110 mg/dL and 4.5 mg/dL, respectively, by hospital day 11, necessitating the initiation of acute intermittent hemodialysis. A kidney biopsy was performed on hospital day 21 due to the lack of renal recovery. The biopsy revealed fibrin deposition and fragmented red blood cells within capillary loops and acute tubular injury consistent with thrombotic microangiopathy (Figure 2). Eculizumab therapy was initiated for the diagnosis of aHUS with a standard weight-based induction dosing regimen of 900 mg weekly for 4 weeks, then receiving maintenance dosing with 1,200 mg biweekly. The patient’s urine output gradually increased during his induction dosing with eculizumab, and serum creatinine obtained prior to each hemodialysis treatment decreased steadily, until hemodialysis was discontinued 6 weeks after the initiation of eculizumab. The patient’s estimated glomerular filtration rate was 45 mL/min/1.73m^2^ at the cessation of dialysis. 

Retrospectively, further studies of the complement system were performed on the patient’s serum sample that had been separated and stored at –80°C at the time of his admission, which revealed normal levels of factor H, factor B, and factor I. Testing was also negative for factor H autoantibodies. Sequencing of *CFH*, *CFI*, *MCP*, *CFB*, *C3*, and *THBD* genes as well as multiplex ligation-dependent probe amplification testing (MLPA) of the factor H-related genes *CFHR1* and *CFHR3* failed to identify any known mutations that have been implicated in the pathogenesis of aHUS. EDTA plasma was not obtained to facilitate testing complement activation fragments such as sC5b-9, Bb, and C3a. 

The patient received 23 doses of eculizumab over a total course of 38 weeks. Given his unrevealing genetic assessment and ongoing requirement for intravenous access placement, the patient and his family elected to discontinue treatment. Since discontinuing eculizumab therapy, his hemoglobin and platelets have remained normal, and his renal function has improved, now with stable chronic kidney disease (CKD) stage II. His ulcerative colitis has also remained well-controlled on 6-mercaptopurine without any recurrent disease. 

### Case 2 

**Figure 3.**
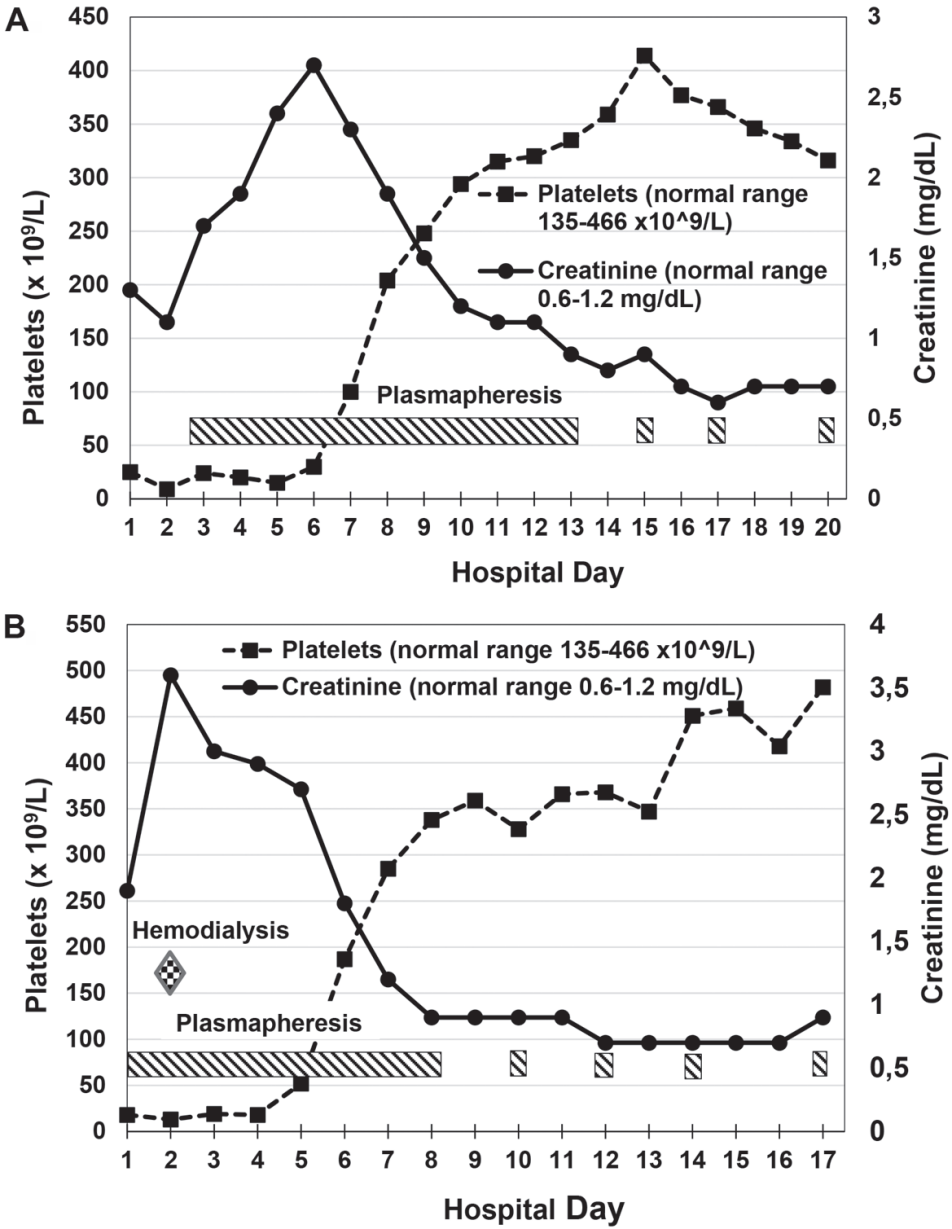
Trends in platelet count and serum creatinine in case 2, both during initial disease presentation (A) and during relapse (B).

A 12-year-old previously healthy Caucasian male presented to the emergency department with a 1-day history of fever, abdominal pain, and acute onset of cola-colored urine. Laboratory testing revealed anemia (Hgb 11.0 g/dL), thrombocytopenia (platelet count of 25 × 10^9^/L), and AKI (serum creatinine of 1.3 mg/dL, BUN of 41 mg/dL). The patient also had a mild coagulopathy, with an INR of 1.98. He was initially diagnosed with disseminated intravascular coagulation (DIC), thought to be secondary to an infectious etiology. However, infectious workup, including heterophile antibody test for EBV, blood and urine cultures, EBV PCR, CMV PCR, and HHV6 PCR, was negative for any known triggers of the DIC. Serum levels of C3 and C4 were normal. Several hours after admission, he developed respiratory distress, and a chest X-ray revealed diffuse bilateral infiltrates consistent with pulmonary edema. Due to the onset of additional end-organ involvement concerning for a multisystem disease, he was treated with TPE for the presumed diagnosis of thrombotic thrombocytopenic purpura (TTP), although pre-TPE ADAMTS13 activity and vWF multimer analysis were later found to be normal. The patient received 11 consecutive daily TPE treatments with fresh frozen plasma (FFP), followed by 3 additional TPE treatments with FFP on alternate days. The patient experienced an anaphylactic reaction during his 4^th^ TPE treatment, but tolerated subsequent treatments without incident. The patient’s thrombocytopenia (Figure 3A) and elevated LDH gradually improved and ultimately normalized by the 9^th^ day of his TPE treatment course. Interestingly, his serum creatinine continued to rise after initiation of TPE (Figure 3A), peaking at 2.7 mg/dL on the 3^rd^ day of TPE treatment, then gradually improved, with renal function normalizing by the 9^th^ day of treatment. 

15 months later, the patient presented to the emergency department again with a 3-day history of fever, abdominal pain, vomiting, diarrhea, and dark cola-colored urine. Initial laboratory testing revealed recurrence of anemia (Hgb of 12.5 g/dL), thrombocytopenia (platelet count of 18 × 10^9^/L/μL), and AKI (serum creatinine of 1.9 mg/dL, BUN of 55 mg/dL). ADAMTS13 activity was again obtained, and later found to be normal, but based on the severe thrombocytopenia and mild renal dysfunction, the patient was given a presumptive diagnosis of relapsed TTP. He received 8 consecutive daily treatments with TPE, followed by 3 additional TPE treatments on alternate days, with gradual improvement and ultimately normalization of his thrombocytopenia by the 5^th^ day of his TPE treatment course (Figure 3B), and his LDH by the 9^th^ day of his course. With this occurrence of his relapsed thrombotic microangiopathy, his renal dysfunction worsened quickly despite initiation of TPE, with creatinine peaking at 3.6 mg/dL (Figure 3B) and BUN peaking at 82 mg/dL and the development of oliguria on the second day of TPE treatment, necessitating a single hemodialysis treatment, then gradually improved, with renal function normalizing by the 6^th^ day of treatment. As ADAMTS13 activity was normal on both occurrences of disease, complement testing performed later revealed normal serum levels of C3, C4, factor H, and factor I but reduced expression of MCP on peripheral blood mononuclear cells by flow cytometry to 52% of a control sample. Subsequent genetic testing revealed a heterozygous mutation (IVS2+2 T>G) in the gene for membrane cofactor protein (MCP), which is known to be associated with aHUS [13]. 

Approximately 2 years later, the patient developed bloody diarrhea and weight loss. He underwent upper endoscopy and colonoscopy that showed colitis with granulomas consistent with Crohn’s disease. He was initially placed on steroids, with resolution of gastrointestinal symptoms. He remains in remission from aHUS and Crohn’s disease without any further immunosuppressant therapy. 

### Case 3 

An 11-year-old Caucasian male with a known history of aHUS originally diagnosed at the age of 6 years was treated previously with multiple episodes of plasma therapy and transitioned to eculizumab according to the manufacturer’s recommendations at the time of a disease relapse associated with a blood catheter infection. His platelet count, hemoglobin, and hematocrit normalized within 30 days of starting eculizumab. The patient’s genetic assessment revealed no mutations in the currently identified genes associated with aHUS. The patient carried an additional diagnosis of iron deficiency anemia. 

After ~ 1 year of eculizumab therapy, the patient presented again with worsening anemia in the absence of overt hemolytic parameters (negative direct Coombs, high haptoglobin, normal platelet count and LDH) but with elevated inflammatory markers (erythrocyte sedimentation rate and C-reactive protein). Additional workup revealed persistence of iron deficiency and worsening of his microcytosis (MCV 71 fL). Due to the worsening anemia without apparent etiology and a modest increase in C5 function indicating breakthrough of his terminal complement blockade despite standard eculizumab dosing, an eculizumab level was tested and found to be in the therapeutic range. No changes in the patient’s anti-complement therapy were made. One month after initial presentation with microcytic anemia, the patient developed diarrhea, with stools positive for occult blood. Upper endoscopy and colonoscopy revealed terminal ileal disease consistent with Crohn’s disease. The patient was placed on mesalamine and infliximab with clinical improvement. 

He remains on a combination of eculizumab, mesalamine, and infliximab, with no evidence of aHUS recurrence. His family history is significant for a brother who also has IBD, but there is no family history of aHUS. 

## Discussion 

The historical terms of “diarrhea positive (D+)” and “diarrhea negative (D–)” HUS were originally meant to distinguish between STEC-HUS and aHUS. However, this terminology has more recently fallen out of favor because it is now understood that diarrhea may be present in as many as 35% of patients with aHUS [14, 15, 16]. As evidenced by these three cases, the presence of bloody diarrhea in cases of HUS may also represent intrinsic gastrointestinal disease and raises many questions about the link between the complement system and gut mucosa. 

Changes in the function of the complement pathway have been previously described in patients with IBD [17, 18, 19]. Such alterations, in the context of a genetic and environmental predisposition to complement dysregulation, could lead to activation or reactivation of aHUS. More recently, Cao et al. showed that human intestinal epithelial cells are a source of complement, as well as are directly affected by activated complement [20]. Additionally, complement factor B has been shown to be upregulated in colonic biopsies of patients with IBD, with an associated increase in complement activation products [21]. Given the considerable amount of antigen exposure in the gut, it would not be unexpected for the gastrointestinal tract to play a large role in triggering systemic complement activation. Such activation may be uncontrolled in patients with an underlying defect in complement regulatory proteins, leading to the clinical manifestations of aHUS. This may explain the sequence of events in which a diagnosis of IBD precedes the clinical onset of aHUS, such as patient 1 in our series and in other patients previously reported with the onset of unspecified HUS following a remote diagnosis of inflammatory bowel disease [8, 11, 12]. 

Conversely, inflammation of the gastrointestinal tract may not only serve as a trigger of aHUS but may result from it as well. As aHUS is a systemic disease causing microvascular thrombosis and injury in both renal and non-renal tissues, loss of gastrointestinal mucosa integrity due to microangiopathy may lead to bacterial translocation and inflammatory changes that occur in IBD. This could explain a reversed sequence of events, in which the diagnosis of aHUS precedes a clinical and/or histopathologic diagnosis of IBD such as patients 2 and 3 in our series. Although any direct benefit of eculizumab in the pathobiology of IBD remains purely speculative, it is interesting to note that with adequate suppression of the complement system in one of the 3 patients reported here, the clinical symptoms of IBD improved markedly despite receiving the same dose of 6-mercaptopurine as he received prior to his diagnosis of aHUS. 

Ultimately, aHUS is still a relatively new entity that is not yet completely understood. A number of genetic mutations have been characterized in this syndrome but still, nearly 40% of aHUS cases lack an identified mutation. Various other disease states, such as HELLP (hemolysis, elevated liver enzymes, low platelet count) syndrome in pregnancy, cancers, and infection with the human immunodeficiency virus, have been linked with aHUS [1]. However, the association between aHUS and IBD in children has not been robustly described before. The relationship between these two disease processes is likely due to a complex interplay between environmental and genetic factors, but further research is needed to better characterize this link. 

## Funding 

All authors declare that they receive no funding support for the work contained within this manuscript. 

## Conflict of interest 

The authors note that they do not have any conflict of interest to declare.
